# Post FDA approval analysis of 200 gallium-68 DOTATATE imaging: A retrospective analysis in neuroendocrine tumor patients

**DOI:** 10.18632/oncotarget.27695

**Published:** 2020-08-11

**Authors:** Aman Chauhan, Riham El-Khouli, Timothy Waits, Rohitashva Agrawal, Fariha Siddiqui, Zachary Tarter, Millicent Horn, Heidi Weiss, Elizabeth Oates, B. Mark Evers, Lowell Anthony

**Affiliations:** ^1^Department of Internal Medicine-Medical Oncology and the Markey Cancer Center, University of Kentucky, Lexington, KY, USA; ^2^Department of Radiology, University of Kentucky, Lexington, KY, USA; ^3^College of Medicine, University of Kentucky, Lexington, KY, USA; ^4^Department of Biostatistics and the Markey Cancer Center, University of Kentucky, Lexington, KY, USA; ^5^Department of Surgery and the Markey Cancer Center, University of Kentucky, Lexington, KY, USA; ^*^Co primary authors

**Keywords:** gallium-68 DOTATATE, neuroendocrine diagnosis, NET

## Abstract

Gallium-68 DOTATATE provides physiologic imaging and assists in disease localization for somatostatin receptor (SSTR) positive neuroendocrine tumor (NET) patients. However, questions regarding usefulness of gallium- 68 DOTATATE imaging in identifying the primary site in neuroendocrine tumors (NETS) of unknown primary, correlation of NET grade with median Standardized Uptake Value (SUV) and effects of long acting somatostatin analog on gallium-68 DOTATATE imaging quality needs to be evaluated.

A single institution retrospective review of the first 200 NET patients with gallium-68 DOTATATE imaging from Dec 2016 to Dec 2017 was conducted. Questions related to NETs of unknown primary, correlation of Standardized Uptake Value (SUV) to Ki-67 (which signifies proliferation rate), the effects of long-acting systemic somatostatin analog (SSA) on SUV were part of our data analysis.

From these 200 patients, 59.5% (119) were females, 40.5% (81) were males; the median age was 62 years. The following primary tumor sites were identified: small bowel-37.5%; pancreas-18.5%; bronchial-14%; colon-3.5%; rectum-2%; appendix-1.5%; adrenal-0.5%; prostate-0.5%; others-3% and unknown primary-19%. Mean hepatic SUV of the lesion with the greatest radiolabeled uptake in 96 patients was similar irrespective to exposure to long acting SSA. Patients exposed to long acting SSA had mean SUV of 31.3 vs 27.8 for SSA naïve patients. The difference was not statistically significant.

Gallium-68 DOTATATE imaging seems to distinguished G3 NET from G1/G2 based on mean SUV, and also identified the primary tumor site in 17 of 38 (45%) patients with unknown primary. Systemic exposure to long acting SSA does not appear to influence mean SUV of gallium-68 DOTATATE scan.

## INTRODUCTION

Neuroendocrine tumors (NETs) are unique neoplasms that are known for their phenotypic as well as molecular heterogeneity [1]. NETs can be classified as functional or nonfunctional based on their ability to produce certain hormones (serotonin, insulin, vasoactive intestinal peptide, gastrin) [2]. NETs can also be characterized as high grade, intermediate grade or low grade based on proliferative activity according to Ki 67 index. Low and intermediate grade well differentiated NETs (Ki 67 ≤ 20%) tend to overexpress somatostatin receptors (SSTRs) and have the potential to secrete various peptide hormones in about 20–40% cases [3]. A Ki 67 index between 3 and 20% is considered intermediate grade or grade 2. The grade 2 NETs tend to progress somewhat rapidly as compared to grade 1, however individual cases can show heterogenous tumor growth rates. Grade 2 NETs also express somatostatin receptors and can produce peptide hormones. Grade 1 and Grade 2 NETs are managed similarly for most part and are by definition well differentiated [4]. High grade neuroendocrine neoplasms are characterized by Ki 67 index of > 20% and are sub-divided into well differentiated and poorly differentiated subtypes [3]. This distinction, based on morphology (well differentiated vs poorly differentiated), is fairly new and strategies regarding distinct managements are currently being explored. In general grade 3 neuroendocrine neoplasms are fast growing, not particularly somatostatin receptor dense, and rarely produce functional hormones.

The overexpression of somatostatin receptors (SSTRs) on NET cell surface has been utilized both diagnostically as well as therapeutically [8, 14]. The ^111^In-pentetreotide scintigraphy (Octreoscan) was the mainstay functional scan to assess somatostatin receptor positivity in low and intermediate grade NETs. Combined with the CT scan, it helped oncologists localize the primary site and accurately assess tumor burden. Due to the general limitation of SPECT (Single Photon Emission Computed Tomography) compared to PET (Positron Emission Tomography) imaging that includes resolution, image quality, and sensitivity, the Octreoscan provided relatively low sensitivity and poor imaging quality. In 2016, FDA approved a new somatostatin receptor analogue PET radiopharmaceutical (gallium-68 DOTATATE) for the diagnosis of well differentiated NETs.

Octreoscan was of great clinical value in the disease analysis, staging, and treatment response assessment for oncologists. However, the value and superiority of gallium-68 DOTATATE has been proven repeatedly in neuroendocrine tumors [13, 14]. Nonetheless real world evidence of application of gallium-68 DOTATATE in post FDA approval era is largely unknown and of great value.

We reviewed over 200 gallium-68 DOTATATE scans and tried to evaluate its role in identifying the primary site for NETs of unknown primary. We also share our single center initial experience with the gallium-68 DOTATATE PET/CT in the USA clinical domain since its FDA approval.

## RESULTS

### Patient population

**Figure 1:**
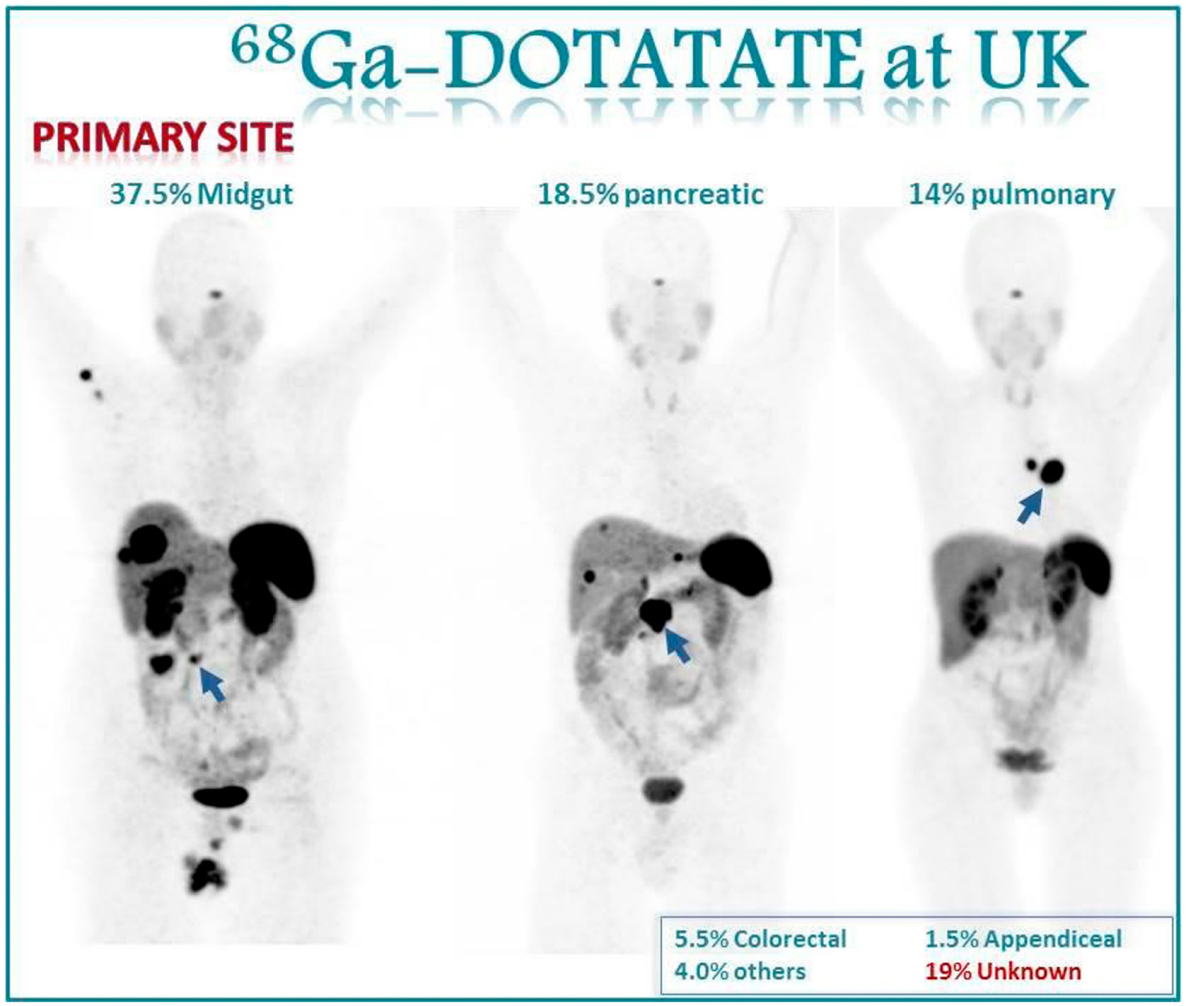
Images show the primary site distribution in our population. Among our first 200 patients who underwent gallium-68 DOTATATE PET/CT scans, 37.5%were midgut primary, 18.5% were pancreatic, and 14% were pulmonary primary. 19% of our cases did not have a primary site identified prior to PET/CT scan. Figure 1 gives examples of how primary midgut, pancreatic and thoracic NETs appear on gallium 68 dotatate imaging.

From these 200 patients, 59.5% (119/200) were females and 60.5% (81/200) were males. The median age was 62 ± 12 (30–84 years). The primary site was known in 81% (162/200) and unknown in 19% (38/200) of patients in our study cohort. Identified primary tumor sites were: small bowel 37.5%; pancreas 18.5%; bronchial 14%; colon 3.5%; rectum 2%; appendix 1.5%; adrenal 0.5%; prostate 0.5%; and others 3% of patients. Additional demographic details are summarized in Table 1. Figure 1 illustrates primary tumor site distribution in our study population. Top 3 sites were identified to be midgut NET, pulmonary NET and pancreatic NET. Figure 1 gives examples of how primary midgut; pancreatic and thoracic NETs appear on gallium 68 dotatate imaging.

**Table 1:** Patient demographics

Demographics	*N* (Percentage) Total = 200
Gender^*^	
** Male**	81 (40.5%)
** Females**	119 (59.5%)
**Median Age^!^**	62+/–12 (30–84)
Primary Site^*^	**Prevalence**
**Small Bowel**	75 (37.5%)
**Pancreas**	37 (18.5%)
**Lung**	28 (14%)
**Colon**	7 (3.5%)
**Rectum**	4 (2%)
**Appendix**	3 (1.5%)
**Adrenal**	1 (0.5%)
**Prostate**	1 (0.5%)
**Others**	6 (3%)
**Unknown Primary**	38 (19%)
Functional Status^*^	**Prevalence**
**Carcinoid Syndrome**	52 (26%)
**No carcinoid Syndrome**	45 (22.5%)
**Functional Status Unknown**	103 (51.5%)

^*^Data represented as number of patients with percentage in parentheses. ^!^Data represented as mean + standard deviation.

**Figure 2:**
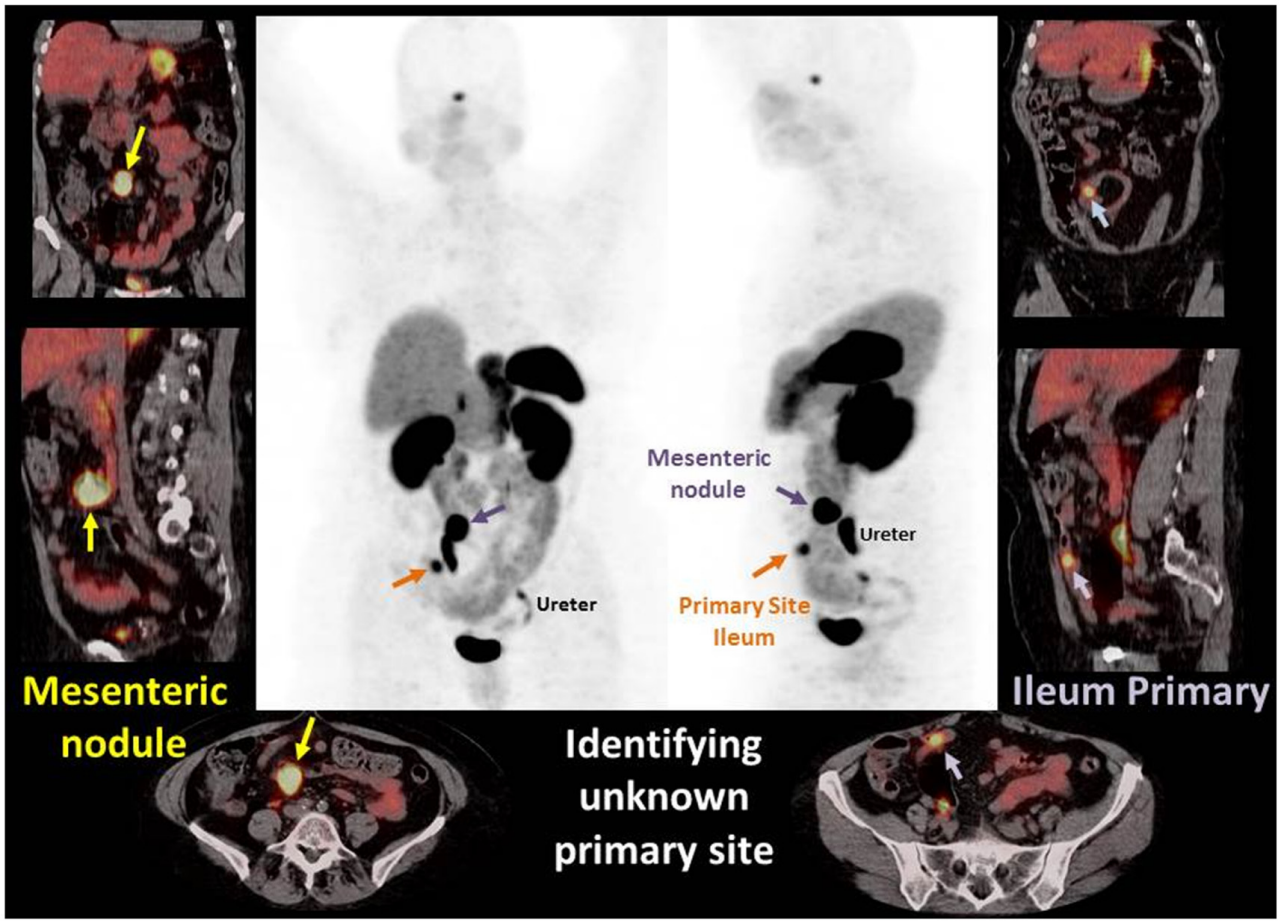
Images demonstrate PET MIP images and fused PET/CT axial, sagittal, and coronal images of a case example of gallium-68 DOTATATE identifying unknown primary site. A 70 year old female patient presented to the emergency department with abdominal pain. Contrast CT of the abdomen and pelvis showed an irregular mesenteric mass and no primary identified. Gallium-68DOTATATE PET/CT demonstrated an avid mesenteric mass and identified an avid ileal primary. Subsequently the patient underwent exploratory laparotomy with small bowel resection and mesenteric node dissection. Pathological examination revealed a well differentiated G2 neuroendocrine tumor. This case highlights capability of gallium 68-dotatate imaging in locating the primary tumor which was otherwise missed in anatomic scans (Contrasted CT scan).

We performed analyses to determine if gallium-68 DOTATATE imaging could identify primary tumors that were otherwise deemed NET of unknown primary. For the purpose of the study we defined the unknown primary as a tumor of whose origin was not established after CT and or MRI scan, upper GI endoscopy and colonoscopy. Within the cohort, 38 patients were diagnosed with NET of unknown primary, and the gallium-68 DOTATATE imaging located primary tumors in 17 of 38 (44%) patients. Figure 2. Images demonstrate PET MIP images and fused PET/CT axial, sagittal, and coronal images of a case example of gallium-68 DOTATATE identifying unknown primary site. A 70 year old female patient presented to the emergency department with abdominal pain. Contrast CT of the abdomen and pelvis showed an irregular mesenteric mass and no primary identified. Gallium-68DOTATATE PET/CT demonstrated an avid mesenteric mass and identified an avid ileal primary. Subsequently the patient underwent exploratory laparotomy with small bowel resection and mesenteric node dissection. Pathological examination revealed a well differentiated G2 neuroendocrine tumor. This case highlights capability of gallium 68-dotatate imaging in locating the primary tumor which was otherwise missed in anatomic scans (Contrasted CT scan).

**Figure 3:**
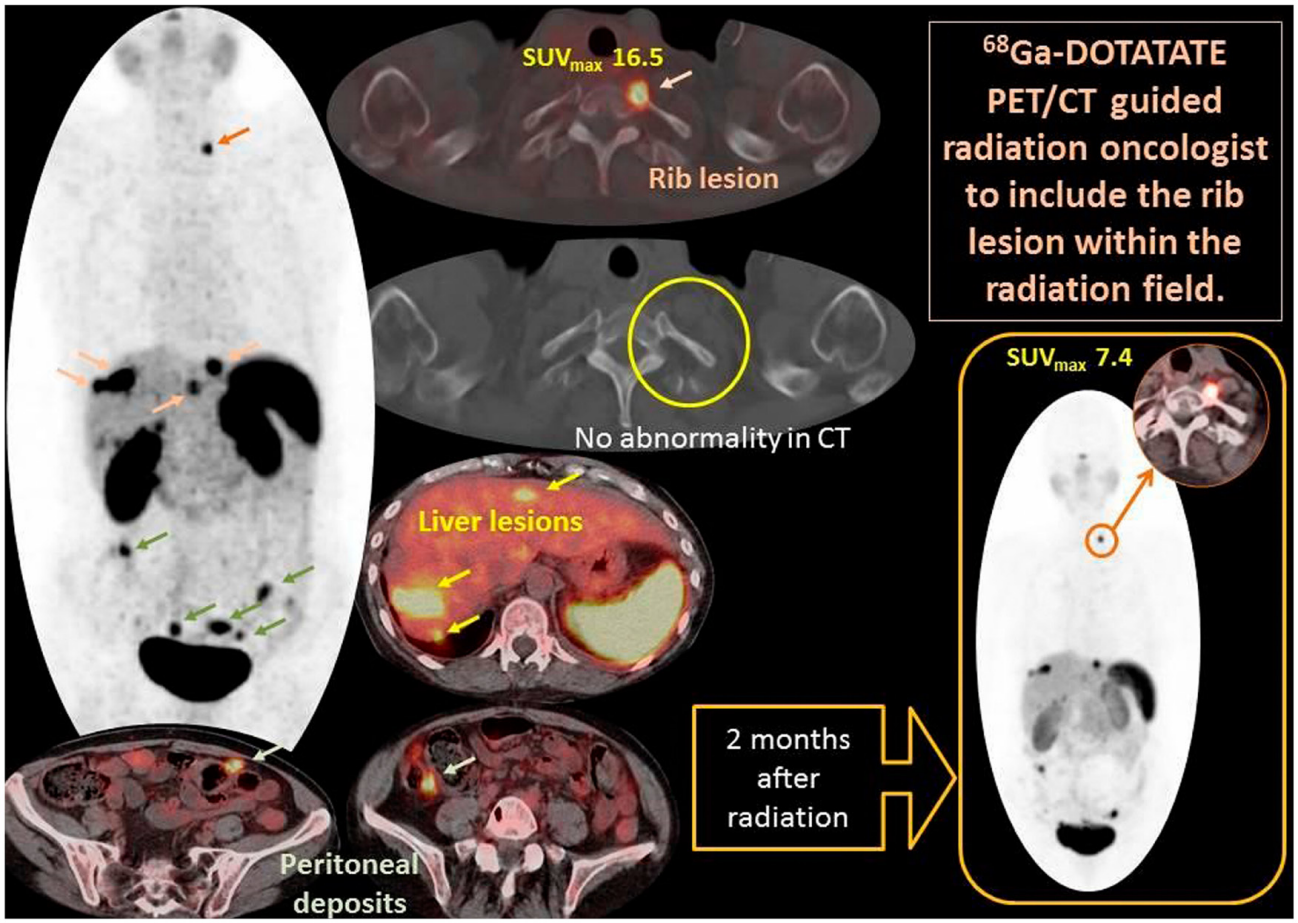
Demonstrates PET MIP image (Central) and fused PET/CT axial images of a case example of gallium- 68 DOTATATE that influenced and guided management. A 69 year old female patient had small bowel well differentiated neuroendocrine tumor metastatic to liver and peritoneum. Gallium- 68 DOTATATE showed intensely avid metastatic focus at the proximal costovertebral end of the left 1st rib (orange arrows), which on clinical correlation was found to cause pain. Case was discussed in multidisciplinary conference and consensus was to proceed with external beam radiation for symptom control. Gallium- 68 DOTATATE PET/CT scan 2 months after completing radiation showed a 55% decrease in uptake (SUV_max_ 7.4 versus 16.5). On the follow up visit patient reported resolution of rib pain after radiation. Gallium 68 dotatate scan was able to pick up rib metastatic lesion and help us formulate plan for palliative external beam radiation resulting in symptom alleviation.

Figure 3 provides an illustrated case study of impact of gallium- 68 DOTATATE on the management. A 69 year old female patient had small bowel well differentiated neuroendocrine tumor metastatic to liver and peritoneum. Gallium- 68 DOTATATE showed intensely avid metastatic focus at the proximal costovertebral end of the left 1st rib (orange arrows), which on clinical correlation was found to cause pain. Of note this lesion was missed on CT scan. Case was discussed in multidisciplinary conference and consensus was to proceed with external beam radiation for symptom control. Gallium- 68 DOTATATE PET/CT scan 2 months after completing radiation showed a 55% decrease in uptake (SUV_max_ 7.4 versus 16.5). On the follow up visit patient reported resolution of rib pain after radiation. Gallium 68 dotatate scan was able to pick up rib metastatic lesion and help us formulate plan for palliative external beam radiation resulting in symptom alleviation.

We also investigated the correlation between somatostatin receptor avidity with the gallium-68 DOTATATE scan and the Ki 67 index as noted on histopathology. Since there was a discordance between the Standardized Uptake Value (SUV) of primary versus metastatic lesions, we focused on the hepatic metastatic lesions to utilize a homogeneous study cohort. Subgroup analysis of mean SUV for hepatic metastatic lesions revealed an uptake of 37.3 for G1 (*n* = 20) as compared to 32.3 for G2 (*n* = 37) and 17.46 for G3 (*n* = 5). Table 2 shows the tumor grade and median SUV of hepatic metastasis with Ga-68 DOTA imaging. Data is suggestive of higher-grade tumor correlating with lower mean SUV. This is due to reduced somatostatin receptor density as tumor grade increases.

**Table 2:** Subgroup analysis of median SUV for hepatic metastatic lesions

Grade 1 (*N* = 20)		Grade 2 (*N* = 37)		Grade 3 (*N* = 5)	
Mean ± SD	37.3 ± 31.91	Mean ± SD	32.3 ± 23.19	Mean ± SD	17.46 ± 25.89

mSUV = maximum standardized uptake value.

Finally, we evaluated the effects of long-acting systemic somatostatin analog (LAR) on the mean SUV. Patients were divided into two cohorts: 41 with primary tumors and 96 with hepatic metastasis. Grade 3 patients were excluded from this analysis since they are not treated with long acting SSA. For this analysis, the newly diagnosed subpopulation of 96 patients, that had never been treated with long acting SSA, were compared with those that were currently treated with long acting SSA. The mean SUV of the most intensely labeled lesions were similar despite exposure to the long acting SSA. The mean SUV of the most intensely labeled metastatic lesion in liver was 31.3 for those using long acting SSA versus 27.8 for those never treated with long acting SSA. Table 3 provides the mean SUV in relation to grade of tumor and presence or absence of LAR. Our findings suggest that exposure to long acting somatostatin analog does not impact mean SUV on gallium 68 dotatate imaging. This is an interesting observation as in clinical practice it is often difficult to co-ordinate long acting somatostatin analog injection with a gallium 68 dotatate scan, especially if patient is being referred to a specialized center.

**Table 3:** No meaningful difference in the mean SUV was noted in hepatic metastasis regardless of presence or absence of systemic long acting somatostatin analogs

	*N*	Mean mSUV
**With SSA**	62.0	35.1 ± 23.3
**Without SSA**	34.0	32.9 ± 25.6

mSUV = maximum standardized uptake value; SSA = somatostatin analogs.

## DISCUSSION

NETs express somatostatin receptors on their cell membrane [5, 6], and a variety of diagnostic and therapeutic agents have been developed that act by targeting the surface somatostatin receptor [7, 8]. Among the many such radiolabeled diagnostic agents, which include Ga-68 DOTATOC and ^68^Ga-DOTANOC, it was gallium-68 DOTATATE that rose to preeminence [9–12]. Although gallium-68 DOTATATE was shown to be superior in terms of sensitivity and specificity, it was not clear if technical superiority would translate into clinical benefit [9]. Hermann et al. prospectively studied the impact of gallium-68 DOTATATE scans on the clinical management of NETs. Two questionnaires were sent to the referring physicians who referred 100 NET patients to UCLA to undergo gallium-68 DOTATATE imaging. The results were profound. Physicians reported that imaging with gallium-68 DOTATATE altered clinical management in 60% patients [13]. Building on these impressive results, Calais et al., applied for an expanded access IND and reported their findings on 130 enrolled patients in 2017. Referring physicians were asked to complete a written questionnaire immediately before the gallium-68 DOTATATE scan and second one upon receipt of the scan report. A follow up third questionnaire was also obtained at 6 months. Information from 96 patients were evaluable. The gallium-68 DOTATATE scan altered clinical decision in 50% patients (48 out of 96 patients) [14]. Prior to gallium-68 DOTATATE PET/CT scan, the primary site was not identified in almost 19% of our population and gallium-68 DOTATATE PET/CT identified the primary site in almost 45% of NETs of unknown primary. Yao and colleagues reviewed the Surveillance, Epidemiology, and End Results (SEER) program registries from 1973 to 2004 to assess the epidemiology of NETs. They reported that among the total 35,825 patients diagnosed with NET within the SEER database, 4,752 (13%) did not have a primary site identified [15]. Our study showed an average percentage of unknown primary site, 19%, which is similar. A study conducted by Sadowski et al. prospectively evaluated NETs of unknown primary and found that gallium-68 DOTATATE PET/CT imaging identified primary tumors in 4 out of 14 patients (28.6%) that were not identified on conventional imaging [16]. A more recent study conducted by Menda, and colleagues studied the value of ^68^Ga-DOTATOC PET/CT scan in 40 patients with metastatic NET but no identified primary site. They reported that ^68^Ga-DOTATOC PET/CT identified primary site in 38% of the cases [17]. These results agree with our data, where ^68^Ga-DOTATOC PET/CT identified the primary NET site in 45% of our cases with unknown primary.

We also evaluated the change in mean SUV on a gallium-68 DOTATATE scan with grade of tumor. As previously stated, NETs are divided into grade 1, 2 and 3 based on Ki 67 index, a marker of proliferative activity. As Ki 67 index increases, the somatostatin receptor density decreases. Kayani et al. reported their findings on 18 neuroendocrine lung neoplasms. In that study, low grade NETs showed intense uptake on gallium-68 DOTATATE scan whereas high grade neuroendocrine neoplasms were FDG avid but had low SUV on the gallium-68 DOTATATE scan [18]. Our results showed consistent trends. Subgroup analysis of mean SUV for hepatic metastatic lesions revealed higher values for G1 as compared to G2 and G3 neoplasms on the gallium- 68 DOTATATE scan (Table 2). And as also seen in Figure 2, a low-grade NET shows avid uptake on a gallium-68 DOTATATE scan.

Finally, we wanted to evaluate the effects of systemic somatostatin analog therapy on the quality of a gallium-68 DOTATATE scan. Theoretically, for optimal quality with a gallium-68 DOTATATE scan it would be preferable to avoid exposure to a long acting release somatostatin analog like LAR. However, in the real world it is often logistically difficult to suspend somatostatin analog prior to the gallium-68 DOTATATE scan; optimally one should coordinate a nadir in LAR levels with a gallium-68 DOTATATE scan. Our hypothesis was that the presence of systemic log acting SSA does not impact quality of gallium-68 DOTATATE scan. To test the hypothesis, we divided our study cohort into two groups: newly diagnosed NETs who were never exposed to the long acting SSA and metastatic NET patients on chronic monthly long acting SSA. Being on monthly SSA will mean a constant therapeutic level of SSA in systemic circulation. Surprisingly, the mean SUV with the greatest uptake were similar in LAR-treated and -naïve patients. It can be cautiously stated that presence of long acting SSA does not significantly alter gallium-68 DOTATATE image quality and hence there is no absolute necessity that long acting SSA be administered only after the scan. Our finding is consistent with recent studies where long acting SSA have been noted to have little or no impact on tumor and metastatic tissue [19, 20].

Limitations of our study include the retrospective design, yet we included the consecutive initial 200 NET patients who underwent ^68^Ga-DOTATOC PET/CT in our institution to prevent selection bias in our data. For sub group analysis we were limited by the small sample size and therefore slightly underpowered study which made it difficult to demonstrate statistically significant differences, however clinically relevant trends were noted as mentioned above.

## MATERIALS AND METHODS

### Patients

Our study was approved by the Institutional Review Board (IRB) and is Health Insurance Portability And Accountability Act (HIPAA) compliant with the consent form waiver. We retrospectively reviewed our first 200 clinical NET patients who underwent gallium-68 DOTATATEPET/CT since FDA approval in June 2016. These patients were managed at University of Kentucky’s Markey Cancer Center from December 2016 to December 2017. Electronic medical records were screened for medical history, pathological diagnosis, radiological data and pertinent laboratory values. We did a subgroup analysis to determine if gallium-68 DOTATATE imaging could identify primary tumors that were otherwise deemed NET of unknown primary. We also investigated the correlation between somatostatin receptor avidity with the gallium-68 DOTATATE scan and the Ki 67 index as noted on histopathology. We evaluated the effects of long-acting systemic somatostatin analog (LAR) on the SUV as a subgroup analysis.

### Technique and image acquisition

According to our clinical protocol, all of our patients are instructed to fast for 4–6 hours and avoid strenuous activity during the 24 hours prior to a scan. Weight based dosage of gallium-68 DOTATATE (1.998 Mbq/kg body weight to a maximum of 259 Mbq) was administered intravenously. After an average of 60 minutes uptake, both PET and CT images were acquired on a 64-detector PET/CT system (Biograph mCT, Siemens Medical Solutions, USA), from vertex to mid-thigh. PET acquisition was performed for 5 minutes per bed position with a 500 mm field of view (FOV), and 168 × 168 matrix. Our institution uses 5 minutes per table position to improve the image statistics, signal to noise, resolution, and overall image quality. We did not observe any significant drop in SUV for our patient population. PET images were reconstructed with an iterative algorithm (ordered-subset expectation maximization; 2 iterations, 8 subsets). CT based attenuation correction and image registration was performed as well. Data required for quantitative analysis and uptake value measurements including patient weight, time of injection, uptake time, and injected activity (syringe activity prior to injection – residual syringe activity after injection) were added to scanning information. Radiolabeling and quality control of gallium-68 DOTATATE were performed by our local radio-pharmacy according to the manufacturer guidelines to avoid any errors or variations.

### Image analysis

Qualitative and quantitative image analyses were performed using MRADA workstation (Reveal-MVS; Mirada Solutions, Oxford, England). Information regarding primary lesion location, metastatic sites location, and lesions Maximum Standardized Uptake Value (SUV_max_) were recorded. Patient management pre and post gallium-68 DOTATATE scan were collected from medical records by an independent investigator who was not involved in clinical management of the patient.

### Statistical analysis

Continuous data were presented as the mean ± standard deviation (SD), while categorical data were presented as number of cases and percentage (%). Nonpaired *t* test was used to compare means and chi square test was used to compare proportions. *P*-value of ≤ 0.05 was considered statistically significant. Analyses were performed using STATA version 15, statistical software (Stata, College Station, TX).

## CONCLUSIONS

We deem the gallium-68 DOTATATE PET/CT to be central in the management of NET patients, that the scan can help in detecting the site of a NET that was previously unknown, and also support tumor grade analysis (low grade vs high grade NETs) and therefore tumor management.

## Abbreviations

CT: computed tomography
FDG: fluorodeoxyglucose
gallium-68 DOTATATE: 68-gallium DOTATATE
NET: neuroendocrine tumor
PRRT: peptide receptor radionuclide therapy
SSA: somatostatin analog
SUV: standardized uptake value
MIP: maximum intensity projections
